# Chronic Folliculitis Associated with *Ovine gammaherpesvirus 2*-Induced Infections in Dairy Cows from Southern Brazil

**DOI:** 10.3390/ani15192883

**Published:** 2025-10-01

**Authors:** Selwyn Arlington Headley, Flávia Helena Pereira Silva, Mariana da Silva Marques, Juliana Torres Tomazi Fritzen, Fernanda Pinto-Ferreira, Geovana Depieri Yoshitani, Ana Aparecida Correa Xavier, Pedro Paulo Benyunes Vieira, Amauri Alcindo Alfieri

**Affiliations:** 1 Laboratory of Animal Pathology, Department of Preventive Veterinary Medicine, Universidade Estadual de Londrina, Londrina 86057-970, Brazil; flaviahelena.pereira@uel.br (F.H.P.S.); ana.xavier@uel.br (A.A.C.X.); 2 Multi-User Animal Health Laboratory (LAMSA), Department of Preventive Veterinary Medicine, Universidade Estadual de Londrina, Londrina 86057-970, Brazil; alfieri@uel.br; 3 Programa de Pós-Graduação em Saúde e Produção Animal, Universidade Pitágoras Unopar Anhanguera, Arapongas 86702-670, Brazil; 4 Laboratory of Animal Virology, Department of Preventive Veterinary Medicine, Universidade Estadual de Londrina, Londrina 86057-970, Brazil; mariana.silva.marques@uel.br (M.d.S.M.); jufritzen@uel.br (J.T.T.F.); geovana.depieri@uel.br (G.D.Y.); 5 Zoonoses and Epidemiology Laboratory, Department of Preventive Veterinary Medicine, Universidade Estadual de Londrina, Londrina 86057-970, Brazil; fpferreira@uel.br; 6 Policlínica Veterinária Pioneiros, Carambeí 84145-000, Brazil; pedrobvieira@gmail.com

**Keywords:** cutaneous disease, diagnostic immunohistochemistry, *Macavirus*, malignant catarrhal fever, viral dermatitis

## Abstract

*Ovine gammaherpesvirus 2* (OvGHV2) is a *Macavirus* and the cause of sheep-associated malignant catarrhal fever (SA-MCF) in susceptible mammalian hosts worldwide. OvGHV2 may produce typical clinical manifestations of SA-MCF or subclinical infections. Additionally, OvGHV2 is associated with cutaneous lesions in ruminants, with few documented reports of this unusual manifestation worldwide. This paper presents the pathological, immunohistochemical (IHC), and molecular findings observed in outbreaks of OvGHV2-related skin infections in dairy cattle from Southern Brazil. Cutaneous scrapings (*n* = 35) and biopsies (*n* = 6) were obtained from dairy cows derived from three farms. All cows (*n* = 35) developed widespread, ulcerative to scaly and erythematous skin lesions, and had no contact with sheep or goats. The biopsies were evaluated for histopathological diagnosis and then used in IHC analyses designed to detect malignant catarrhal fever virus (MCFV) antigens and to evaluate the inflammatory response. All scrapings and biopsies were used in PCR assays to amplify OvGHV2. Additionally, all biopsies were used in PCR assays to detect *bovine gammaherpesvirus* 6 (BoGHV6), *bovine alphaherpesvirus 1* (BoAHV1), and poxvirus. Histopathology revealed chronic folliculitis in all biopsies. IHC detected intralesional, intracytoplasmic MCFV antigens in most (83.3%; 5/6) of the cutaneous lesions with folliculitis. These skin lesions showed a strong T-cell response, macrophage clusters, and caspase-positive follicular keratinocytes. OvGHV2 DNA was detected in 66.7% (4/6) of the cutaneous biopsies that contained MCFV antigens and in 8.6% (3/35) of the cutaneous scrapings. The DNA of BoGHV6, BoAHV1, and Poxvirus was not amplified from any of the cutaneous biopsies. These findings demonstrated that OvGHV2 was associated with the cutaneous lesions in dairy cows at these farms and represent the first description of OvGHV2-related skin disease in ruminants from Brazil and the entire Latin America. A review of previous cases of skin lesions associated with infections by OvGHV2 revealed that most cases had a histological diagnosis of folliculitis, suggesting that folliculitis may be associated with OvGHV2-related skin infections. Additionally, this investigation contrasts all previous reports of OvGHV2-related skin disease in ruminants, since the infected cows herein identified were not reared concomitantly or within proximity of the asymptomatic reservoir host. Furthermore, the possible form of OvGHV2 dissemination to the susceptible cows during this study is discussed.

## Simple Summary

This study investigated outbreaks of skin infections in dairy cattle from Southern Brazil caused by *Ovine gammaherpesvirus 2* (OvGHV2). This virus is the cause of sheep-associated malignant catarrhal fever, with associated skin lesions having been rarely identified in affected animals worldwide. Cutaneous scrapings and biopsies were obtained from affected cows that had widespread ulcerative and erythematous skin lesions. Histopathological analysis showed chronic folliculitis, while antigens of malignant catarrhal fever virus were detected in most lesions by immunohistochemistry. OvGHV2 DNA was found in a significant portion of the biopsies, while other viruses were not detected. The infected cows were not reared with or near sheep (asymptomatic reservoir hosts), contrasting with all previous reports of OvGHV2-related skin infections identified worldwide. This indicates that there may be other transmission mechanisms. A review of previous cases suggests that folliculitis may be associated with OvGHV2-related skin infections in ruminants. These findings represent the first description of OvGHV2-related skin disease in ruminants from Brazil and the entire Latin America, expanding the geographical occurrence of this unusual viral skin disease.

## 1. Introduction

*Ovine gammaherpesvirus* 2 (*Macavirus ovinegamma* 2; OvGHV2) is a member of the *Macavirus* genus, *Gammaherpesvirinae* subfamily [1]. OvGHV2 is one of the few well-investigated *Macavirus* that causes malignant catarrhal fever (MCF) in a wide range of mammalian hosts [2,3,4]. This *Macavirus* is a member of the MCF virus (MCFV) complex due to sharing of the 15A antigenic epitope [5] and being well conserved at the genetic level with other MCF viruses [6]. *Acephaline gammaherpesvirus* 1 (AlGHV1) is a *Macavirus* that is antigenically related to OvGHV2 and produces MCF predominantly in ruminants from Africa [3,7]. Alternatively, the *Macavirus, bovine gammaherpesvirus 6* (BoGHV6), was never associated with the development of MCF, since it is unknown if this virus shares the 15A antigenic isotope [8]. Nevertheless, BoGHV6 was identified in ruminants from Brazil [8,9,10,11], North America [12,13,14], Europe [15,16,17], and New Zealand [18], but the capacity to develop diseases in animals is considered controversial [8] or without any effect [19]. Currently, OvGHV2 and BoGHV6 were the only *Macavirus* detected in ruminants from Brazil, with OvGHV2 being associated with MCF in this continental nation [4].

Animals infected with OvGHV2 may or may not develop clinical manifestations of disease resulting in typical recognizable syndromes of sheep-associated malignant catarrhal fever (SA-MCF). This is because subclinical infections due to OvGHV2 relative to clinical manifestations of disease may be more prevalent in specific geographical locations [20,21,22]. Nevertheless, infections by OvGHV2 produce a wide spectrum of disease syndromes typical of SA-MCF in susceptible mammalian populations worldwide [2,4,7]. These clinical syndromes include the “head and eye” form [2,3,4,7], and the alimentary [3,4], neurological [4,7], and cutaneous [3,7] manifestations of SA-MCF. OvGHV2 induces a predominantly T-cell-mediated inflammatory response in affected animals [7,23,24].

The “head and eye” form of SA-MCF is the most frequently diagnosed clinical manifestation of OvGHV2-related infections [3,7], and is characterized principally by abrupt fever, nasal discharge, corneal opacity, enlarged lymph nodes, and ulcerations of the alimentary tract [3,4]. Alternatively, there are comparatively few descriptions of cutaneous lesions associated with OvGHV2 infections in mammalians worldwide. This scarcity may suggest that this form of OvGHV2-infection is a rare manifestation, that could have been confused with skin lesions of ruminants, or underdiagnosed. Although sheep and goats infected by MCFV may develop a non-pruritic scaling and crusting disease that affects several anatomical locations of the affected animal [25], there are few descriptions of cutaneous manifestations of OvGHV2-related infections in mammals worldwide. Additionally, all the published data originated from predominantly Europe and North America, with individual reports from Oceania and Asia. In these cases, infections were associated with granulomatous mural folliculitis (GMF) in goats from the USA [26,27], the UK [28], and Belgium [29], and in a sika deer from the UK [30]; crusting alopecia in a cow from New Zealand [31], cervids in the USA [32], a big-horn sheep from Canada [33], and a goat from the USA [34]. Additional related cutaneous manifestations associated with infections by OvGHV2 included erythema multiforme in a goat from Switzerland [35], chronic dermatitis in cows from the UK [36], systemic necrotizing vasculitis in sheep from Ireland [37], and cutaneous ulcerations in cattle from Israel [36]. Reports of cutaneous manifestations of OvGHV2-related infections were not located from Latin or South America when major English and Latin databases were examined. Consequently, the objectives of this study were the following: (1) to present the pathological, immunohistochemical, and molecular findings observed in outbreaks of cutaneous manifestations associated with OvGHV2-related infections in dairy cattle from Southern Brazil, and (2) to provide additional evidence of the association between OvGHV2 and follicular inflammation in ruminants.

## 2. Materials and Methods

### 2.1. Study Location, Animals, and Sampling

The outbreaks of cutaneous lesions occurred in dairy cattle reared within the municipalities of Carambeí and Ponta Grossa, Paraná, Southern Brazil, from January to December 2023. These two municipalities are located within the Central–Eastern mesoregion of the state of Paraná, Atlantic biome. The estimated ruminant population of Carambeí in 2023 was 56,715 heads of cattle and 1227 heads of sheep, while in Ponta Grossa, there were 29,878 heads of cattle and 5480 sheep [38]. Cattle within this mesoregion consisted predominantly of Holstein and Jersey breeds reared exclusively for commercial milk production. Carambeí was considered the second-largest milk producer within the state of Paraná and contributed with 269.9 million Liters in 2023 [38]. Most of the farms within these municipalities are closed dairy units, i.e., there has been no recent introduction of animals to these farms over the last 15–20 years. Additionally, sheep and goats were not reared on these farms or within proximity. Curiously, 5.9% (6/100) of cows reared in five closed dairy establishments within the municipality of Carambeí had detectable antibodies of OvGHV2 [20].

Farms A and B were located within the Northwestern and Southeastern regions, respectively, of the municipality of Carambeí, while Farm C was within the municipality of Ponta Grossa. There were several reported episodes of non-pruritic cutaneous lesions at these dairy farms: the first occurred in January-February, then May, followed by October, and lastly, in December 2023. Farm A consisted of 1800 cows, Farm B had 1200 heads, and Farm C had 400 cows. However, only lactating dairy cows (i.e., primiparous or cows that recently gave birth) were affected at each farm, indicating that all outbreaks occurred during the transition period of dairy cows [39,40]. During each outbreak, the cutaneous lesions had a reported evolution of approximately 40–50 days, being resolved after pulverization with a wide-spectrum disinfectant (Virkon-S^®^, Lanxess, São Paulo, Brazil).

All samples were surgically incised and submitted by the consulting veterinarian for these dairy establishments to determine the possible cause. Cutaneous scrapings (*n* = 35) and biopsies (*n* = 6) from cows with skin lesions and maintained at these three dairy farms were received for laboratory analyses. All samples were randomly collected by the consulting veterinarian as the cutaneous lesions appeared at these farms. The sampling periods as well as the number and type of samples obtained from each farm are summarized in Table 1. All samples received were maintained at −80 °C until used in molecular analyses. However, cutaneous biopsies were only obtained from cows at Farms B and C.

### 2.2. Pathological Evaluations

All cutaneous biopsies were routinely immersed in 10% buffered formalin solution and processed for histopathological evaluation with the Hematoxylin and eosin stain. Selected formalin-fixed paraffin-embedded (FFPE) tissue sections were used for the identification of fungal organisms by the Grocott histochemical stain, for the immunohistochemical (IHC) detection of MCFV antigens, and to evaluate the inflammatory response.

### 2.3. Immunohistochemical Identification of MCFV Antigens in Dairy Cows with Folliculitis

Immunohistochemical analyses were performed using the 15A-MAb IHC assay, standardized to detect intralesional antigens of MCFV [41] from selected FFPE tissue sections. Positive control consisted of the utilization of FFPE sections known to contain OvGHV2 antigens from previous studies [22,41]. Negative controls consisted of replacing the 15A-MAb with its diluent and the utilization of the 15A-MAb on FFPE sections known to demonstrate negative immunoreactivity for OvGHV2. Negative and positive controls were included in all IHC assays.

### 2.4. Immunohistochemical Characterization of Inflammatory Reaction in Cutaneous Samples of Dairy Cows

Immunohistochemical (IHC) assays were used to investigate the possible inflammatory response and the participation of apoptosis in the development of cutaneous lesions in these dairy cows. Accordingly, a panel of immunological markers was used to determine the extent and distribution of T (CD3) and B (CD79a) lymphocytes, macrophages, and apoptosis (Casp3) within the cutaneous fragments evaluated. A list of the immunological markers with the associated clones, dilutions, methods of antigen retrieval, and information from the manufacturers is provided in Table 2.

### 2.5. Molecular Characterization of Ovine gammaherpesvirus 2 Tegument Protein Gene and Macavirus from Cutaneous Samples

Two nucleic acid extraction protocols were used. The DNA extraction from most samples was performed with the automated extraction system Loccus Extracta 32 (Loccus do Brasil Ltd.a. São Paulo, Brazil) using the commercial DNA extraction kit (Extracta Kit DNA and RNA pathogens MDX, Loccus do Brasil), as recommended by the manufacturer. However, the DNA extractions from samples collected during March 2023 were performed using a combination of the phenol/chloroform/isoamyl alcohol and silica/guanidine isothiocyanate protocols, as described [42,43].

The extracted nucleic acids were then used in semi-nested PCR (snPCR) assays designed to detect the partial fragment of the OvGHV2 tegument protein gene, ORF75 [44], the BoGHV6 glycoprotein B gene [45], bovine alphaherpesvirus 1 (BoAHV1) glycoprotein C gene [46], and the RNA polymerase gene of Poxvirus [47]. Additionally, samples that contained MCFV antigens by IHC but did not contain OvGHV2 or BoGHV6 DNA by the PCR assays were used in molecular assays designed to detect a broad spectrum of herpesviruses [48,49]. A list of the target genes, primers, and the amplicon sizes of the agents used during this investigation is provided (Supplementary Table S1).

The positive control for the ORF75 assay consisted of OvGHV2 DNA derived from previous studies of clinical SA-MCF in cattle from Brazil [50,51]; for the BoGHV6 and BoAHV1 PCR assays, DNA from previous studies was used [8]. Sterile ultrapure water was used as the negative control in all nucleic acid extractions. All PCR products were separated by electrophoresis in 2% agarose gels, stained with ethidium bromide, and examined under ultraviolet light.

### 2.6. Sequence Determination of the OvGHV2 Tegument Protein Gene

The products derived from the molecular assays were purified with the commercial Wizard SV Gel and PCR ClenUp System (Promega^®^ Life Technologies, Madison, WI, USA), quantified using a commercial Fluorometer (Qubit^®^ Invitrogen^®^ Life Technologies, Eugene, OR, USA), and then submitted to direct sequencing in both directions with the forward and reverse primers used in the OvGHV2 snPCR assay in an ABI3500 Genetic Analyzer sequencer (Ibaraki-ken, Japan).

Sequence quality analyses and consensus sequences were obtained using PHRED and CAP3 homepage (http://asparagin.cenargen.embrapa.br/phph, accessed on 18 October 2024), respectively. Thereafter, nucleotide (nt) similarity searches were performed to identify nt ORF75 sequences deposited in GenBank using the BLAST homepage (https://blast.ncbi.nlm.nih.gov/Blast.cgi, accessed on 18 October 2024).

Sequence alignment and identity matrix were performed by using the BioEdit software, version 7.7.1. [52]. The nt sequence derived from this study was then compared with reference strains of OvGHV2, AlGHV1, and BoGHV6 and with strains of OvGHV2 identified in ruminants from different biomes of Brazil and from other geographical regions. The evolutionary history was inferred using the Neighbor-Joining method [53] and the distances were computed using the Kimura 2-parameter method [54], using the MEGA7 software [55] (Version 7.0).

### 2.7. Types of Macavirus Associated Infections

The characterization of the type of infection observed in each cow was determined due to the combination of the IHC detection of tissue antigens of MCFV and the molecular amplification of OvGHV2 DNA in the same animal. Consequently, a diagnosis of OvGHV2-related infections was established due to the detection of MCFV tissue antigens by IHC with the concomitant amplification of OvGHV2 DNA by snPCR. Alternatively, an infection was termed MCFV-associated with cows that did not contain either OvGHV2 or BoGHV6 DNA by PCR but demonstrated positive immunoreactivity with the 15A-MAb IHC assay.

### 2.8. Possible Association of Wild Boars with the Occurrence of OvGHV2-Induced Cutaneous Lesions in Dairy Cows

These dairy farms were in the Central–Eastern mesoregion of Paraná state where free-ranging wild boars (*Sus scrofa*), subclinically infected with OvGHV2 were identified [22], and an association was established between these roaming animals and the seropositivity to OvGHV2 antibodies in dairy cows reared in closed herds [20]. Accordingly, we wanted to evaluate the possible association of these wild boars on the occurrence of OvGHV2-induced skin lesions in cattle that had no contact with sheep or goats.

A buffer zone was established to associate the movement of free-ranging wild boars with the occurrence of skin lesions associated with OvGHV2 based on previous studies that estimated the distance travelled by free-ranging wild boars. One study demonstrated that the estimated distance travelled by wild boars varied between 76 km (average 1 km/day) during the resting (sedentary) phase and 310 km (average 6 km/day) during the dispersion phase [56]. Alternatively, another investigation demonstrated that male wild boars could travel for an average of 5.86 km/day, with females traveling 5.13 km/day [57]. Additionally, the home range of free-ranging wild boars may vary between 33 to 87 km^2^ [58]. Therefore, we have established a home range of 50 km for the movement of free-ranging wild boars [20].

To this effect, a 50 km buffer, considering the daily roaming activity of wild boars relative to their home range [20,58], was established using the locations where these wild boars were captured to evaluate the possible occurrence of cutaneous lesions (positive/negative) between dairy farms located inside and outside of the geographic buffer.

The statistical analysis was performed using the Epi Info™ 7.2 software (https://www.cdc.gov/epiinfo, accessed on 2 September 2025 [59], using the Chi-square test (χ^2^) without continuity correction; when an expected frequency was <5, the Fisher’s exact test was automatically used. A significance level of 5% (*p* < 0.05) was adopted. To quantify the association, the odds ratio was calculated with a 95% CI. The spatial evaluation was conducted by mapping the area of the three farms from this study and the locations where all known subclinically infected free-ranging wild boars were captured [21,22] using the QGIS software, version 3.28. The coordinates were georeferenced with the SIRGAS 2000/UTM Zone 22S reference system (EPSG:31982), compatible with the study area. Input data for the utilization of wild boars subclinically infected by OvGHv2 were derived from previous studies [21,22]. The properties were plotted as a layer of vector points, and areas of influence were delimited using a buffer with a radius of 50 km.

### 2.9. Statistical Analyses

The association between the type of cutaneous sample (biopsy or scraping) and OvGHV2 detection by snPCR was assessed using the online software OpenEpi (version 3.01; http://www.openepi.com, accessed on 2 September 2025 [60]. The two-tailed Fisher’s exact test was applied, as it is appropriate for small sample sizes. The odds ratio (OR) and 95% confidence interval (95% CI) were calculated. Additionally, exact binomial 95% confidence intervals for proportions (Clopper–Pearson method) were calculated to indicate the precision of infection rates, especially in groups with small sample sizes. *p*-values < 0.05 were considered statistically significant.

## 3. Results

### 3.1. Clinical and Epidemiological Information

The affected cows at each dairy farm had no clinical manifestations suggestive of SA-MCF. The clinical signs were restricted to the cutaneous lesions; pruritus or fever was not observed. The morbidity of the cutaneous disease observed in the dairy cows varied from 1.3% (20/1500) within the municipality of Carambeí to 20% (80/400) in dairy cows from the municipality of Ponta Grossa (Table 1).

The rates of the OvGHV2-related infections, considering both biopsy and cutaneous scrapings, observed at each by dairy farm are provided at Table 1. Infection was comparatively more elevated at Farm C (50%; CI, 1.3–98.7), relative to Farms A (9.5%; CI, 1.2–30.4), and B (18.8%; CI, 4.1–45.7). These results reflect the variability and uncertainty of the analyses due to the relatively reduced number of samples evaluated.

The comparison of the diagnostic efficiency between biopsy and skin scrapings for the detection of OvGHV2 by snPCR in dairy cows with cutaneous lesions is presented in Table 3. The positivity rate for OvGHV2 by snPCR was significantly higher (*p* = 0.0095) in biopsy samples (4/6; 66.7%) as compared with skin scrapings (3/35; 8.6%). Furthermore, the chances of detecting OvGHV2 DNA were 21x higher (Table 3) with biopsy samples (Odds Ratio = 21.33; 95% CI: 1.85–289.7) as compared with skin scrapings.

### 3.2. Gross, Histopathological, and Immunohistochemical Findings

**Figure 1:**
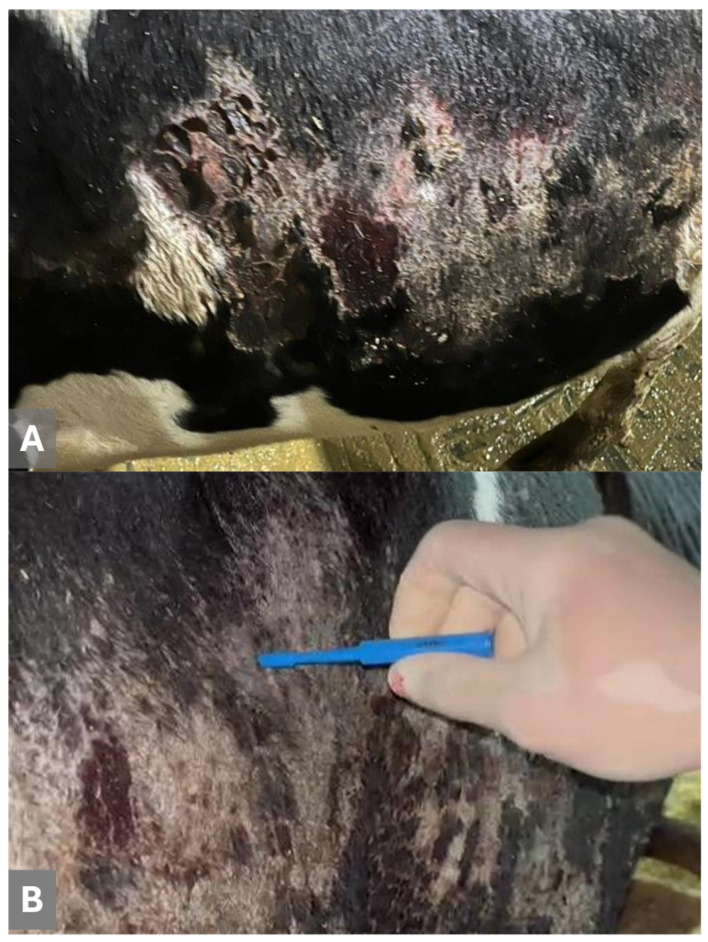
Gross demonstration of the cutaneous lesions observed in dairy cows infected by *ovine gammaherpesvirus 2.* Observe the erythematous, crusting, scaly lesions on the flank of a cow (**A**); a closer view is provided (**B**).

The gross lesions were more severe in dairy cows from the municipality of Carambeí relative to those from Ponta Grossa. The cutaneous lesions observed in the dairy cows were widespread, ulcerative to scaly, with irregular patches of erythema, and occurred predominantly at the flanks of the affected cows (Figure 1A,B).

**Figure 2:**
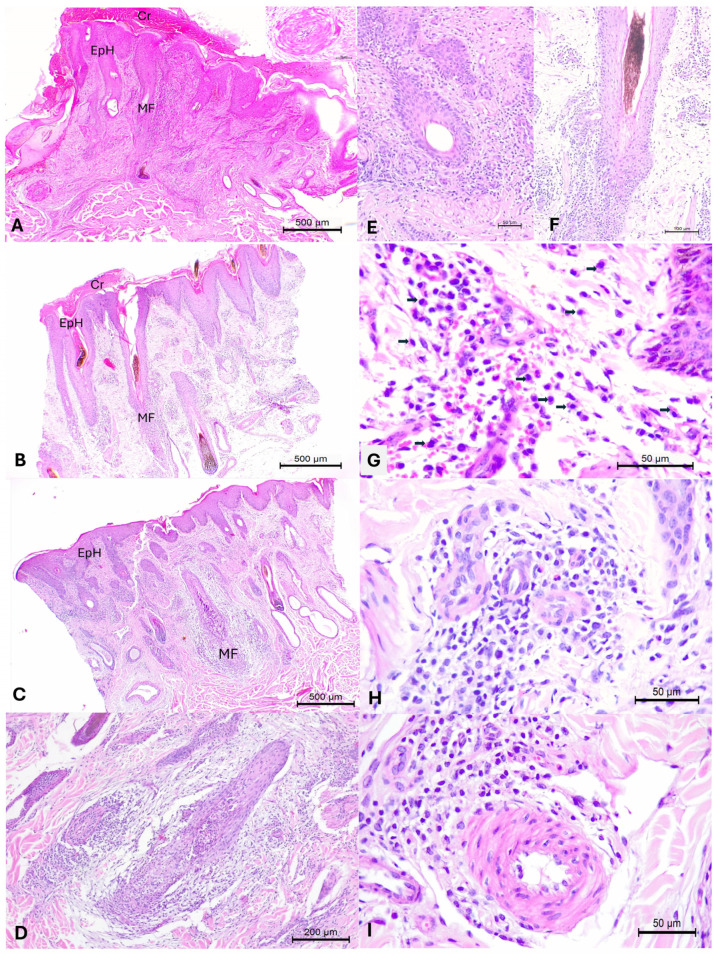
Histopathological findings observed in the skin of dairy cows infected with *Ovine gammaherpesvirus 2*. There is epidermal hyperplasia (EpH) and crusting exudate (Cr) at the surface of the skin (**A**,**B**); observe endothelial proliferation of an artery that is better appreciated with the insert (**A**), and varying degrees of mural folliculitis (MF; **A**–**C**). The difference in the severity of the follicular lesion is easily recognized at the closer images (**D**–**F**). Observe the severe accumulations of macrophages (arrows) within the dermis (**G**), and the chronic lymphoplasmacytic perivasculitis affecting small (**H**) and medium-sized (**I**) dermal vessels. Hematoxylin and eosin stain. Bars, (**A**–**C**) 500 µm; (**D**) 200 µm; (**E**,**G**–**I**) 50 µm; (**F**) 100 µm.

The principal histological alterations observed are summarized in Table 3; with alterations being comparatively more severe in dairy cows from Farm C relative to those from Farm B. Histopathology (Figure 2) revealed severe irregular epidermal hyperplasia with orthokeratotic hyperkeratosis with the accumulation of a purulent crusting exudate. The extent and severity of the inflammatory reaction within the underlying dermis varied between the fragments evaluated. However, most contained accumulations of mononuclear cells, consisting predominantly of macrophages, with varying populations of lymphocytes and plasma cells, and some eosinophils.

In all cutaneous fragments evaluated, the hair follicles were affected. In some cases, there was mild accumulation of inflammatory cells around hair follicles, while in others, there was severe inflammatory reaction at the follicular epithelium with subsequent mural folliculitis and destruction of hair follicles. In some animals, there were areas of perivasculitis involving small and medium-sized vessels, while in others, there was marked endothelial proliferation resulting in partial obliteration of the vascular lumen. Additionally, in some animals, there was moderate to severe lymphocytic inflammation of the sebaceous glands adjacent to hair follicles. Fungal organisms were not identified with the Grocott histochemical stain.

**Figure 3:**
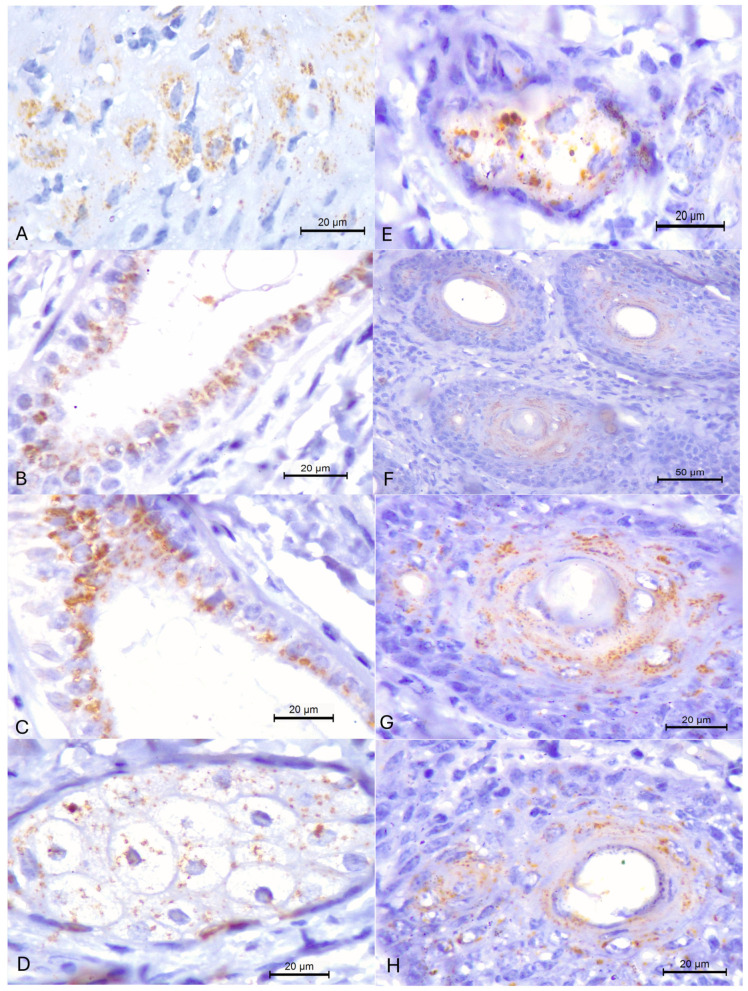
Immunohistochemical detection of MCFV antigens in dairy cows with chronic folliculitis. There is positive intracytoplasmic immunoreactivity within epithelial cells of the epidermis (**A**), apocrine (**B**,**C**) and sebaceous (**D**,**E**) glands, and follicular keratinocytes (**F–H**). Immunoperoxidase counterstained with Hematoxylin. Bars, (**A**–**E**,**G**,**H**) 20 µm; (**F**) 50 µm.

The 15A-IHC assay, designed to identify MCFV antigens, revealed positive intracytoplasmic immunoreactivity in most (83.3%; 5/6) of the dairy cows evaluated from Farms B and C with a histopathological diagnosis of folliculitis (Table 4). Positive intracytoplasmic immunoreactivity (Figure 3A–D) was observed within the epithelial cells of the sebaceous (80%; 4/5) and apocrine glands (60%; 3/5), and within follicular keratinocytes (60%; 3/5) as well as in the squamous epithelium of the skin. In some hair follicles, the hair shaft was destroyed, but there was widespread positive immunoreactivity within follicular keratinocytes. MCFV antigens were not detected in the cutaneous fragments of cow #4 from Farm B. The positive and negative controls used in each IHC assay are provided (Supplementary Figure S1).

### 3.3. Inflammatory Response Observed in Cutaneous Lesions of Dairy Cows Infected by OvGHV2

**Figure 4:**
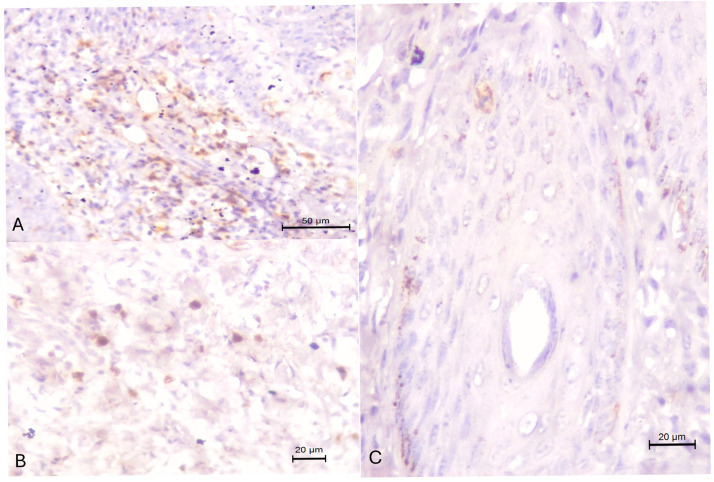
Inflammatory response observed in the cutaneous lesions of cows spontaneously infected by *Ovine gammaherpesvirus 2*. Observe the detection of T-lymphocytes (**A**) and macrophages (**B**) within the dermis, and cleaved caspase within follicular keratinocytes (**C**). Immunoperoxidase counterstained with Hematoxylin. Bars, (**A**) 50 µm, (**B**,**C**) 20 µm.

There was positive detection of CD3-positive lymphocytes (Figure 4A) and macrophages (Figure 4B) within the dermis and positive immunoreactivity to cleaved caspase, particularly within follicular keratinocytes (Figure 4C).

### 3.4. Molecular Detection of OvGHV2 and Macavirus in Cutaneous Samples

The snPCR amplified the desired base pairs of the ORF75 gene from most (66.7%; 4/6) of the cutaneous lesions evaluated with histological demonstrations of folliculitis (Table 2), as well as in 8.6% (3/35) of the cutaneous scrapings from dairy cows at each farm (Table 1). The strain derived from this study was named OvGHV2/BRA-UEL/PR-687/2023 and is deposited in GenBank (accession # PQ613580). Additionally, there was the concomitant amplification of the OvGHV2 ORF75 gene in three of the cows that contained MCFV antigens by IHC, confirming that the tissue antigens detected were OvGHV2.

However, in cows #1 and 6 there was the positive detection of MCFV antigens without the amplification of either OvGHV2 or BoGHV6 DNA, indicating that these animals were probably infected by another *Macavirus*. Accordingly, two types of infections were observed: (a) OvGHV2 associated with folliculitis in cows #2, 3, 4, and 5, and (b) a MCFV-induced folliculitis in cows #1 and 6.

The DNA of BoGHV6, BoAHV1, and Poxvirus was not amplified from any of the samples evaluated. Additional herpesviruses were not detected with the primers designed to amplify a wide range of herpesviruses from mammalian tissues [48,49].

### 3.5. Phylogenetic Analyses of the OvGHV2 Tegument Protein Gene

The OvGHV2 strain derived from this study had 100% nt sequence homology with strains derived from cows (OR761839; MZ221210) reared within the same geographical region of Brazil, as well as in a cow from South Africa (EU851177), and the complete OvGHV2 genome derived from a sheep (DQ198083.1). Additionally, this strain herein identified had 99% nt sequence identity with the OvGHV2 reference strain (NC007646) and with a goat (OK490363) from Southern Brazil. Furthermore, this strain from the cows with cutaneous lesions had 99.5% nt sequence identity with similar strains identified in cows from the Cerrado (KJ658293; KJ658294) and Caatinga (JQ780444; JQ78044) biomes of Brazil and with a cow from Turkey (JN991056). Alternatively, this strain herein detected had 66.5% nt sequency identity with the prototype AlGHV1 strain and 67.8% sequency identity with the BoGHV6 reference strain.

**Figure 5:**
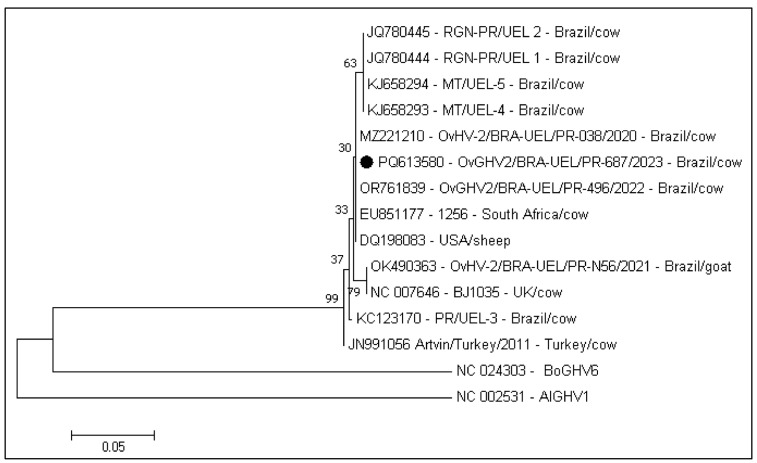
Phylogenetic tree based on selected strains of *Macavirus* tegument protein gene. The percentage of replicate trees in which the associated taxa clustered together in the bootstrap test (1000 replicates) are shown next to the branches. The tree is drawn to scale, with branch lengths in the same units as those of the evolutionary distances used to infer the phylogenetic tree. The analysis involved 14 nucleotide sequences; the strain derived from this study is highlighted (•). The evolutionary analyses were conducted in MEGA7 software.

The phylogenetic evaluation revealed three somewhat distinct clusters of OvGHV2 strains from distinct geographical locations (Figure 5), with the prototype AlGHV1 and BoGHV6 strains being separated from all strains of OvGHV2. The strain derived from this investigation clustered with strains identified in cows from the same geographical region of Brazil as well as that obtained from a South African cow and the complete genome sequence from a sheep. Curiously, the OvGHV2 strains identified in cows from the Cerrado and Caatinga biomes of Brazil were within the same cluster. The third cluster contained the OvGHV2 reference strain as well as strains detected in cows from Brazil and Turkey.

### 3.6. Spatial Association of Free-Ranging Wild Boars on the Occurrence of Skin Lesions in Adult Cows from Closed Dairy Farms

**Figure 6:**
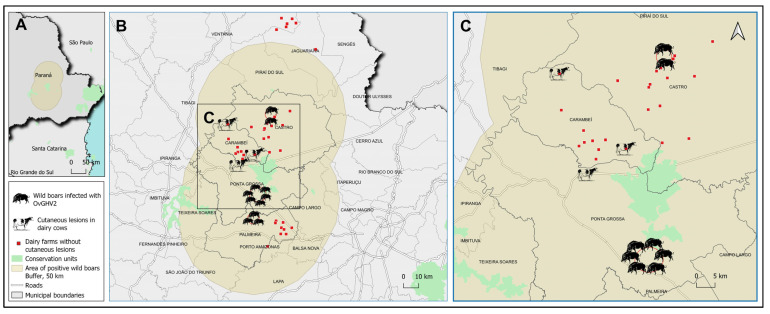
Spatial distribution of the localization of the dairy cows with skin lesions induced by OvGHV2. An overview of the study location in Southern Brazil is provided (**A**). The geographical localization of dairy cows with skin lesions, the region of capture of the free-ranging wild boars, and the dairy farms without skin lesions relative to the 50 km buffer is shown at (**B**), while a closer view of the spatial distribution is given at (**C**).

Within the area delimited by the 50 km buffer, three dairy farms had cows with cutaneous lesions caused by OvGHV2-associated infections. In the other 30 farms similar cases were not identified (Figure 6). However, there were no dairy farms with cutaneous lesions outside of the buffer zone. Accordingly, no significant statistical (*p* = 0.213) association by the Chi-square test (χ^2^) was observed between the localization of the dairy farms and the occurrence of cows with the cutaneous lesions. Additionally, there was no significant (*p* = 0.541) relationship by the Fisher’s exact test.

## 4. Discussion

The results of this study represent the first confirmation of OvGHV2-related skin diseases in cattle from Brazil and Latin America. Thus far, skin-related infections induced by OvGHV2 have been described in cows (*n* = 3), goats (*n* = 6), a sika deer, and two sheep; all are mammals from diverse geographical locations (Table 5). Furthermore, sheep are reservoirs for OvGHV2 [2,4], and these hosts normally do not demonstrate clinical manifestations of infection. A review of previous reports of OvGHV2-associated skin disease in ruminants revealed that most cases had prolonged clinical evolution, varying from 2 months to more than 4 years (Table 5). The previously described cases of OvGHV2-induced dermatitis in cows had reported evolutions that varied from 2.5 [31] to 4 [36] months; the current cases at the three dairy farms occurred for approximately 40 days. At these farms, the affected animals were pulverized with a wide spectrum disinfectant, after which the lesions receded. Although the manufacturer indicated that the disinfectant had viricidal action, there is no documented evidence to support this assertion.

Collectively, these findings may suggest that the gross appearance of the skin disease associated with OvGHV2 infections may be directly related to the period of evolution, varying from mild erythematous lesions, as observed in this study, through alopecia with cutaneous crusts in bighorn sheep [33], to severe crusting formations as occurred in goats [26]. Therefore, it seems as if the gross appearance of OvGHV2-related dermatitis in ruminants may be time-dependent and without any specific gross presentation of the cutaneous disease, which makes the clinical diagnosis a challenge for practicing field veterinarians.

The low morbidity of OvGHV2-associated cutaneous lesions in this investigation is consistent with the epidemiological trends of infections due to OvGHV2 and/or SA-MCF [2,4,7]. The histopathological findings confirmed that the cutaneous manifestations observed in all dairy cows evaluated from Farms B and C were consistent with those of folliculitis; similar findings were reported in goats infected with OvGHV2 [26], suggesting that folliculitis may be a histological feature of ruminants that may be associated with the cutaneous manifestation of OvGHV2 (see below). Furthermore, the histological pattern of skin disease observed in these cases is characteristic of chronic manifestations of cutaneous injury [25]. In three of the six cows evaluated with cutaneous lesions and histological evidence of folliculitis in the current study, OvGHV2 antigens and DNA were detected by IHC and snPCR, respectively, confirming the association of this virus with the cutaneous lesions observed during this outbreak.

Furthermore, the phylogenetic evaluation, based on ORF75, demonstrated that the OvGHV2 strain associated with the cutaneous lesions herein described has elevated nt identity with the reference strain as well as with strains of OvGHV2 identified in cattle from different biomes of Brazil and in ruminants from other countries; similar results were previously described [61,62,63]. However, since this gene is well conserved [64], a better understanding of the evolutionary relationships between strains of OvGHV2 from distinct geographical locations requires the analysis of specific loci of this virus [65].

OvGHV2 DNA was detected in 8.6% (3/35) of the cutaneous scrapings from all three farms, corroborating with the detection of OvGHV2 within the skin biopsies and the association of this pathogen with the skin lesions herein described. Moreover, the chances of the amplification of OvGHV2 in biopsy samples derived from cows with skin lesions were 21.3× (CI 95%, 1.85–289.7) more elevated relative to cutaneous scrapings. The comparatively low detection of OvGHV2 in cutaneous scrapings relative to biopsies may suggest that cutaneous scrapings were not adequate samples for the confirmation of OvGHV2-related infections. In two cows with folliculitis, there was the detection of MCFV antigens by IHC without the amplification of OvGHV2 or BoGHV6 DNA, suggesting the participation of another *Macavirus.* Similar findings were described in MCFV-associated infections in cattle from Brazil [63,66,67]. In the current investigation, efforts to determine the MCFV detected by the 15A-IHC assay were unsuccessful (see below), since no herpesvirus was amplified by two distinct molecular assays [48,49] designed to identify a wide range of herpesvirus in animals. These results demonstrated the complexity associated with the molecular detection of gammaherpesvirus in mammalian tissues worldwide [68].

An interesting feature during this investigation was the accumulations of mononuclear inflammatory cells around dermal vessels; similar findings were observed in goats [26] and sheep [33] with GMF and in a goat with erythema multiforme [35]. Moreover, OvGHV2 mRNA was detected in several cellular populations in the goat with erythema multiforme, including T-lymphocytes, macrophages, vascular endothelia, and keratinocytes [35]. These findings may indicate that the dermal and vascular lesions are probably viral related and part of the known pathogenesis of OvGHV2 [2,3], with consequent viral dermatitis.

### 4.1. The 15A-MAb IHC Assay Can Be Used to Detect OvGHV2 Antigens in Organs of Ruminants

During this investigation MCFV antigens were detected in most (60%; 4/6) of the cutaneous biopsies of cattle with skin lesions with the 15A-IHC assay. This assay was standardized to detect OvGHV2 antigens in cattle with SA-MCF [41], based on the 15A Monoclonal antibody (15A-MAb), initially used in serological assays [69,70]. Additionally, confirmation that the *Macavirus* herein detected was indeed OvGHV2 was obtained due to the concomitant amplification with direct sequencing of the PCR products from 75% (3/4) of the cutaneous lesions that contained MCFV antigens. Furthermore, phylogenetic analyses revealed that the OvGHV2 strain associated with the skin lesions has similar nt sequence identity with the reference strain and other wild type strains of OvGHV2 circulating in Brazil and other geographical locations. Similar results using a combination of the 15A-IHC assay with the simultaneous molecular amplification of OvGHV2 DNA was conducted to confirm OvGHV2-associated infections in cattle [66,67,71,72,73], a goat [62], sheep [61,74], and wild boars [22]. Moreover, this IHC assay was previously used to detect intralesional OvGHV2 antigens in fetal organs of sheep [61] and cattle [63]. Since this IHC assay detects the 15A epitope which is located at the glycoprotein B gene [75] and is shared among all *Macavirus* [5] known to be associated with the development of MCF, the demonstration of the specific *Macavirus* identified must be confirmed by another diagnostic method, such as PCR, as was conducted during this study. Therefore, a specific IHC assay for the detection of OvGHV2 antigens in animals without the concomitant utilization of a complementary diagnostic assay is required.

In two cases during this investigation, a diagnosis of MCFV-related infections was obtained due to the non-amplification of OvGHV2 and BoGHV6 DNA from animals with histological evidence of cutaneous disease and the intralesional detection of MCFV antigens by the 15A-IHC assay. This form of characterization of *Macavrius*-related infections is recommended with the utilization of this specific monoclonal antibody in association with molecular testing, considering that the 15A epitope is shared between all MCFV known to develop MCF in their respective hosts [5,76]. This is because there is demonstrated cross-reactivity between MCFV with the 15A-MAb [70,76], while reactivity with common herpesviruses of ruminants was not observed [70]. In the specific cases herein described, attempts to identify the associated *Macavirus* were frustrated, since PCR assays designed to amplify a wide spectrum of herpesviruses in mammalian tissues [48,49] revealed negative results. Similar findings of MCFV-associated infections were described in cattle [63,66,67] and wild boars [22] from Brazil, where it was suggested the possibility of the circulation of an undiagnosed *Macavirus* in mammalians from this continental nation. Furthermore, simultaneous infections by more than one *Macavirus* were previously described in the same animal [6,77], which becomes a diagnostic challenge with the utilization of consensus primers. Although the utilization of consensus primers has been used for the identification of novel gammaherpesvirus [78], pitfalls were identified with the use of broad spectrum primers for the identification of unknown herpesvirus in mammalian tissues: (a) pan-herpes PCR assays may not be able to amplify all herpesvirus in the same sample if there are several concomitant abundant strains, and (b) the amino-acid motifs used to derive the nucleotide sequence during primer design primers are not conserved in all herpesvirus species and are encoded by highly degenerated codons, resulting in primers that are unable to bind with the same affinity to DNA polymerase genes from different herpesvirus species [68,79]. These two factors could have contributed to the non-amplification of herpesvirus (see below) by consensus PCR assays during this investigation.

Although the exact reason for the non-amplification of herpesvirus during this study with the diagnostic assays using consensus primers are unknown, several factors could have been responsible for these results, including the presence of a very distant member of the *Macavirus* genus in the tissue not detectable by these consensus primers or the non-amplification by the consensus PCR assays of adequate copies of DNA to be observable in the agarose gel [63]. Moreover, the detection of simultaneous occurring herpesvirus within the same samples may not be detectable by PCR with consensus primers [79]. Furthermore, an extremely reduced viral load present in skin biopsies could have also been associated with the non-detection by the consensus primers. In addition, the identification of unrecognized gammaherpesvirus is complex and would require the utilization of multiple strategies [68]. Additionally, it must be highlighted that consensus primers or pan-PCR assays are not perfect for the amplification of all organisms contained within a particular biological sample, since they may demonstrate low specificity and sensitivity when used to detect distantly related genes or when there is low viral load in the sample that is being evaluated [79]. Consequently, the identification of unknown *Macaviruses* continues to be a diagnostic challenge in Brazil and elsewhere, and the utilization of alternative diagnostic methods, including different consensus primers, and innovative methodologies, such as Long-Distance PCR Assay and primer walking [68], are being considered.

### 4.2. Folliculitis May Be Associated with OvGHV2-Related Skin Infections in Ruminants

The confirmation of OvGHV2-associated folliculitis in these dairy cows represent the first description of the association of this pathogen with cutaneous diseases of ruminants from Brazil, and all Latin and South America, considering that all previous cases of skin-related manifestations of OvGHV2 in mammalians occurred predominantly in Europe [27,28,29,30,35,37], and North America [5,26,33], with single reports from Israel [36] and New Zealand [31]. Accordingly, there are relatively few confirmed published cases of OvGHV2-associated cutaneous manifestations worldwide, when compared with the clinical syndromes of SA-MCF. Although the exact reason for the paucity of reports of cutaneous-related manifestations associated with OvGHV2 relative to other common forms of SA-MCF is unknown, underdiagnosis or misdiagnosis may be the likely explanations. Firstly, as indicated above, there seems to be no definite pattern for the gross manifestations of OvGHV2-induced skin lesions in ruminants, which makes a clinical diagnosis difficult and challenging. Secondly, OvGHV2 or *Macavirus* is not included in the differential etiologic diagnosis of animals with cutaneous disease by veterinarians, including diagnostic pathologists, worldwide. This fact was demonstrated by the utilization of diagnostic exercises to emphasize the occurrence of OvGHV2-related cutaneous lesions in farm animals [27,34], so that consulting pathologists can include this agent in cases of skin lesions of farm animals, particularly when there is vasculitis or perivasculitis with folliculitis.

During this investigation, all six cows had histological manifestations indicative of chronic folliculitis, with OvGHV2 antigens being identified within the follicular keratinocytes of some animals; similar findings were described in goats with GMF [26,28] and erythema multiforme [35] associated with infections by OvGHV2, suggesting that this viral pathogen was associated with the development of folliculitis. Additionally, positive immunoreactivity to caspase was detected within follicular keratinocytes of the cows with chronic folliculitis, indicating that apoptosis or necrosis, via the caspase pathway, may be part of the pathogenesis associated with the development of OvGHV2-induced folliculitis in ruminants. Necrosis, as observed during this investigation, was previously associated with experimental infections by OvGHV2 in rabbits [23,24]. Alternatively, GMF with concomitant infection by OvGHV2 was diagnosed in one goat, in which it was proposed that the granulomatous cutaneous inflammatory reaction may be immune-mediated and not directly induced by the viral pathogen [29]. Furthermore, OvGHV2-induced cytotoxicity of infected keratinocytes was associated with the development of GMF and cytotoxic interface dermatitis in a goat [28]. Consequently, the pathogenesis of dermatitis associated with OvGHV2 infections in ruminants seems ill-defined. Therefore, additional cases of this unusual manifestation and experimental infections are needed to fully understand the underlying factors related to the development of these skin lesions in ruminants.

In this case, the absence of multinucleated giant cells within the inflammatory reaction prevented a histological diagnosis of GMF. Nevertheless, GMF was one of the most frequently described patterns of cutaneous manifestations due to OvGHV2, occurring in 45.5% (5/11) of all previous reports [26,27,28,29,33]. Additionally, folliculitis was part of the histological description of goats with cutaneous disease associated with infection by OvGHV2 [34,35]. Taken together, folliculitis was described in 66.7% (8/12) of all known reported cases of OvGHV2-associated dermatitis in ruminants worldwide. These findings may suggest that this histological feature of cutaneous disease may be used to associate OvGHV2 with skin infections in mammals. Notwithstanding the above, the number of skin-related OvGHV2 infections diagnosed worldwide is too small to determine the pattern of skin lesion or the exact pathogenesis without experimental studies. However, since these cutaneous lesions can be confused with other frequently occurring skin diseases of ruminants, it is recommended that this virus be included in the differential diagnosis of ruminants with cutaneous disease in which there is some form of folliculitis, and particularly, vasculitis.

### 4.3. Alternative Forms of Transmission Should Be Considered for the Dissemination of OvGHV2 to the Terminal Hosts in Regions Where These Are Not Reared Concomitantly with Sheep

The current study is unique in that it represents the only reported occurrence of OvGHV2-associated skin lesions in ruminants without any known contact with either sheep or goats. This is because these farms are enclosed high-producing dairy units without the introduction of new animals during the past 15–20 years, and there is no rearing of sheep nor goats at these farms or within proximity. Consequently, how dairy cows became infected by OvGHV2 at these three farms is not completely elucidated. Nevertheless, all cows with the reported skin disease were lactating dairy cows with infections occurring during the transition period; cutaneous lesions also occurred during the transition period in the dairy cow from New Zealand [31]. During the transition period of dairy cows, there are numerous physiological, metabolic, and nutritional activities that frequently results in a wide range of metabolic disorders that are stress-related [40], which make these cows more susceptible to infections [39,40]. Although intense stress could have had some role in the development of these skin lesions at these dairy farms, particularly in cows from Farm A (see below), where seropositivity to OvGHV2 antibodies was previously detected [20]. The actual demonstration of stress was not evaluated during this investigation, so it is unknown of the possible effects of stress during the transition phase relative to the occurrence of these skin lesions. Additionally, immunosuppression due to concomitant bacterial and parasitic infections was considered as a possible trigger mechanism for the development of the skin disease in the bighorn sheep [33], probably due to a latent infection, since this ruminant species is an adapted reservoir host for OvGHV2 [2,3].

Another issue that does not favor the normal dissemination of infection during this study was the low sheep/cattle ratio (SCR) for these municipalities, being estimated at 0.02 for Carambeí and 0.18 for Ponta Grossa. This then suggests that the densities of these ruminant populations within the state of Paraná cannot maintain the active circulation of OvGHV2 between the reservoir and susceptible hosts to result in clinical manifestations of SA-MCF [80]. The SCR is an estimate of the population of sheep to cattle reared within a particular geographical region to evaluate the possible occurrence of clinical outbreaks of SA-MCF in Rio Grande do Sul Southern Brazil [80], where these ruminant species are traditionally maintained on the same pastures [81] and the most reported number of clinical outbreaks of SA-MCF were reported in Brazil [4]. Although reported clinical outbreaks of SA-MCF within the state of Paraná are scarce, there is an increase in the number of cattle diagnosed with subclinical infections due to OvGHV2 in Brazil [20]. Therefore, the epidemiology of clinical outbreaks of SA-MCF and subclinical infections associated with OvGHV2 in Brazil is fascinating and warrants investigation.

A serological evaluation, using samples collected in 2019, detected OvGHV2 antibodies in dairy cows from the municipality of Carambeí, including one asymptomatic adult dairy cow from Farm B [20]. Interestingly, three of the four biopsy samples and one cutaneous scraping from dairy cows at this farm contained OvGHV2 DNA, demonstrating that infection occurred in cows maintained within a herd with seropositivity to OvGHV2 antibodies; sampling for the current study was performed during 2023. Curiously, seropositivity to OvGHV2 antibodies was not detected in the dairy cows tested from Farm A [20], where OvGHV2 DNA was amplified in 4.8% (1/21) of the cutaneous scrapings evaluated during this study. Additionally, we had postulated that possible cow-to-cow transmission was most likely responsible for the seropositive reaction to OvGHV2 antibodies in enclosed dairy farms without contact with either sheep or goats [20]. In-herd transmission between populations of bison and buffalo was also proposed to explain the occurrence of OvGHV2 infections in bison [82] and buffalo [83] that had no prior contact with sheep. Accordingly, it is reasonable to suggest that the dairy cows from Farm B had prior contact with OvGHV2 antibodies and probably developed cutaneous manifestations associated with this pathogen due to the stress of the transitional period of lactating dairy cows, in which the occurrence of infectious disease is more elevated [39].

Inquisitively, all cows with skin lesions associated with infections by OvGHV2 and/or MCFV were reared in dairy farms that were within the home range of free-ranging wild boars that were subclinically infected by OvGHV2 [21,22]. However, no statistical association between the occurrence of OvGHV2-induced folliculitis or vial dermatitis at these dairy farms and the presence of free-ranging wild boars was established during this study, suggesting that dairy cows at these farms were not at risk of developing skin lesions. Caution must be taken with the interpretation of these results, since the risk of occurrence of cutaneous infection during this study was partially affected by the relatively reduced number of dairy farms with this specific manifestation of OvGHV2 when compared to dairy farms without skin lesions. This proportional disparity affected the robustness of the statistical and spatial analyses. Nevertheless, cows with cutaneous lesions from Northwestern Carambeí were located approximately 25 km from the site where asymptomatic free-ranging wild boars infected with OvGHV2 were found, while those from Northeastern Carambeí were situated 20 km away from these wild boars. Additionally, dairy cows from the municipality of Ponta Grossa were identified 20 km from the capture site of wild boars. Therefore, all dairy cows were within the home range of wild boars during the known resting (76 km) or dispersal (310 km) phases of these animals [56]. Since wild boars may serve as a potential bridge-host for the dissemination of OvGHV2 to susceptible bovine populations maintained in geographical regions where neither sheep nor goats are reared concomitantly with cattle [20], it is plausible to suggest that these animals could have been associated with the dissemination of this pathogen to these closed dairy farms with the subsequent occurrence of viral dermatitis. Additional unusual potential sources suggested for the dissemination of OvGHV2 to susceptible mammalian populations in the absence of sheep included airborne spread or mechanical transmission by birds [84]. Therefore, there is cumulative evidence to indicate that the dissemination of OvGHV2 to susceptible mammalian populations does not necessarily require the known adapted host but can occur under special circumstances in the absence of sheep.

### 4.4. Study Limitations

The evaluation of a larger number of skin biopsies from cattle with cutaneous lesions and from different geographical regions or biomes within Brazil could have provided a better understanding of the epidemiology and infectivity of OvGHV2. However, the paucity of skin infections associated with OvGHV2 in cattle prevented the obtention of samples from different geographical regions and/or biomes of Brazil. Nevertheless, these findings are important and fundamental to understand the dissemination of OvGHV2 in Southern Brazil and represent the first description of OvGHV2-related cutaneous lesions in cattle from all Latin America. Additionally, the inability to effectively characterize the MCFV associated with the cutaneous lesions observed in two animals despite the utilization of consensus herpesvirus PCR assays remains a challenge for the diagnosis of *Macavirus*-related infections in Brazil.

## 5. Conclusions

An investigation was conducted to identify the possible cause of skin disease observed in cows from three dairy farms in Southern Brazil. All infections occurred during the transition period of dairy cows. Malignant catarrhal fever virus (MCFV) antigens were detected in the cutaneous biopsy of six dairy cows with a histopathological diagnosis of chronic folliculitis. Additionally, OvGHV2 DNA was amplified in four cows with folliculitis and with intralesional MCFV antigens detected by IHC and in a comparatively reduced number of cutaneous scrapings. Phylogenetic analysis revealed that the strain of OvGHV2 associated with the skin lesions herein described has elevated nucleotide sequence identity with the prototype and other strains of OvGHV2. These findings demonstrated that the MCFV antigens identified in some of these cows with chronic folliculitis were those of OvGHV2, confirming that this pathogen was associated with the cutaneous disease observed in cows at these dairy farms, and represent the first description of skin disease associated with OvGHV2-infections in ruminants from Brazil and Latin America.

## Figures and Tables

**Table 1:** Epidemiological data with immunohistochemical and molecular findings observed in dairy cows with cutaneous lesions from Paraná state, Southern Brazil.

Dairy Farms	Sampling Periods	Geographical Locations	Cattle		Type of Cutaneous Sample		Identification		Infection Rate (%) (CI, 95%)
			Total	Morbidity	Scrapings	Biopsy	MCFV IHC ^1^	OvGHV2 snPCR ^2^	
A	January	Northwestern Carambeí	1800	25 (1.4%)	13	NC	ND	1	7.7
	May–October				2	NC	ND	0	0
					6	NC	ND	0	0
**Total**					**21**			**2**	**9.5 (1.2–30.4)**
B	February	Southeastern Carambeí	1500	20 (1.3%)	5	NC	ND	0	0
	May–October				7	NC	ND	1	14.3
					NC	4	3	3	75
**Total**					**12**	**4**		**3**	**18.8 (4.1–45.7)**
C	December	Ponta Grossa	400	80 (20%)	2	2	2	1	50.0 (1.3–98.7)

Legend: ^1^. MCFV-IHC: Malignant catarrhal fever virus immunohistochemical assay; ^2^. OvGHV2 snPCR: *Ovine gammaherpesvirus 2* seminested PCR assay.

**Table 2:** List of antibodies, dilutions, method of antigen retrieval, and manufacturer details used in the immunohistochemical assays.

Antibody (Clone)	Dilution	Antigen Retrieval	Source
CD3 (UCHT1)	1:100	Citrate buffer (pH 6.0)	Thermo Fisher Scientific; Rockford, IL, USA
CD79a (HM47)	1:100	Citrate buffer (pH 6.0)	Thermo Fisher Scientific; Rockford, IL, USA
Macrophage (LN-5)	1:100	Citrate buffer (pH 6.0)	Zymed Laboratories Inc; San Francisco, CA, USA
Cleaved Caspase-3 (Asp175)	1:200	Citrate buffer (pH 6.0)	Cell Signaling Technology; Danvers, MA, USA

**Table 3:** Comparison of the diagnostic efficiency of OvGHV2 by snPCR between biopsy and skin scrapings derived from dairy cows with cutaneous lesions from Paraná state, Southern Brazil.

Sampling Method	Animals		*p*-Value	OR (CI 95%)
	Infected/Total	(%)		
Biopsy	4/6	66.67	0.0095	21.33 (1.85–289.7)
Skin scrapings	3/35	8.57		

**Table 4:** Histopathological, immunohistochemical, and molecular findings observed in skin lesions of dairy cows infected with *Macavirus*.

Animal #	Principal Histopathological Diagnoses	MCFV-IHC Immunoreactivity	OvGHV2 snPCR ^1^	Types of Infection
Farm B				
1	Chronic folliculitis with epidermal hyperkeratosis and endothelial proliferation	Squamous epithelium; hair follicle epithelium; sebaceous gland	−ve	MCFV
2	Chronic folliculitis with epidermal hyperkeratosis	Squamous epithelium; hair follicle epithelium; apocrine and sebaceous glands	+ve	OvGHV2
3	Chronic folliculitis with epidermal hyperkeratosis and endothelial proliferation	Hair follicle epithelium	+ve	OvGHV2
4	Chronic folliculitis with epidermal hyperkeratosis	Not detected	+ve	OvGHV2
Farm C				
5	Chronic mural folliculitis with epidermal hyperkeratosis and inflamed sebaceous glands	Squamous epithelium; apocrine and sebaceous glands	+ve	OvGHV2
6	Chronic mural folliculitis with epidermal hyperkeratosis, inflamed sebaceous glands, and perivasculitis	Squamous epithelium; apocrine and sebaceous glands	−ve	MCFV

Legend: ^1^, OvGHV2 snPCR: *Ovine gammaherpesvirus 2* semi-nested PCR assay; +ve, positive; −ve, negative.

**Table 5:** Reported cases of *Ovine gammaherpesvirus 2*-related skin infections in ruminants worldwide.

Affected Animal	Clinical Evolution	Contact Animal	Geographical Location	References
Bighorn sheep	Not reported	Not required	North America	[33]
Cow	4 months	Proximity to sheep	Asia	[36]
Cow	2.5 months	Sheep	Oceania	[31]
Cow	40 days	None	South America	This study
Goat	2 months	Goat	Europe	[27]
Goat	2 weeks–4 years	Sheep	North America	[26]
Goat	3 months	Sheep	North America	[5]
Goat	9 days	Not reported	Europe	[35]
Goat	2 weeks	Sheep	Europe	[29]
Goat	Several weeks	None	Europe	[28]
Sheep	4 months	Not required	Europe	[37]
Sika deer	Not reported	Goat	Europe	[30]

## Data Availability

The nucleotide sequence of the OvGHV2 identified during this study is deposited in GenBank (https://www.ncbi.nlm.nih.gov/genbank, deposited on 31 July 2025).

## Supplementary Materials

The following supporting information can be downloaded at: https://www.mdpi.com/article/10.3390/ani15192883/s1, Figure S1: Positive (A) and negative (B) controls used in all immunohistochemical assays with the 15A-IHC assay. Immunoperoxidase counterstained with Hematoxylin. Bars, 20 µm; Table S1: List of primers, diagnostic strategy, and the genomic targets used during this investigation [44,45,46,47,48,49].
