# Supplementary material for: Chronic Folliculitis Associated with Ovine gammaherpesvirus 2-Induced Infections in Dairy Cows from Southern Brazil
Source: Animals (Basel). 2025 Oct 1;15(19):2883. doi: 10.3390/ani15192883 (PMC12523531; doi:10.3390/ani15192883)
Supplement: Supplementary file 1 [file animals-15-02883-s001.zip › Supplementary Table S1.pdf]

Supplementary Table S1. List of primers, diagnostic strategy, and the genomic targets used during this investigation.

Organisms	Diagnostic strategy	Primers names	Genomic target	Amplicon size (bp)	Reference
Ovine gammaherpesvirus 2	snPCR	556/755/555	Tegument protein gene (ORF75)	238	Baxter et al. 1993
Bovine gammaherpesvirus 6	snPCR	HgB990R/HgB636F/HgB892R	Glycoprotein B gene	257	Kubiś et al. 2013
Bovine alphaherpesvirus 1	PCR	B1/Bcon	Glycoprotein C gene	354	Claus et al. 2005
Poxvirus (high GC)	PCR	high-GC F/high-GC R	RNA polymerase subunit gene	630	Li et al. 2010
Gammaherpesvirinae subfamily	PCR	GammaFWD1/GammaREV1	DNA polymerase gene	650	Okoh et al.2023
Herpesviridae family	snPCR	DFA/ILK/TGV/IYG/KG1	DNA polymerase gene	215 to 315	VanDevanter et al. 1996

References

45. Baxter, S.I.; Pow, I.; Bridgen, A.; Reid, H.W. PCR detection of the sheep-associated agent of malignant catarrhal fever. *Arch Virol* **1993**, *132*, 145-159, doi:https://doi.org/10.1007/BF01309849.

46. Kubiś, P.; Materniak, M.; Kuźmak, J. Comparison of nested PCR and qPCR for the detection and quantitation of BoHV6 DNA. *J Virol Methods* **2013**, *194*, 94-101, doi: https://doi.org/10.1016/j.jviromet.2013.08.006.

47. Claus, M.P.; Alfieri, A.F.; Folgueras-Flatschart, A.V.; Wosiacki, S.R.; Médici, K.C.; Alfieri, A.A. Rapid detection and differentiation of bovine herpesvirus 1 and 5 glycoprotein C gene in clinical specimens by multiplex-PCR. *J Virol Methods* **2005**, *128*, 183-188, doi:https://doi.org/10.1016/j.jviromet.2005.05.001.

48. Li, Y.; Meyer, H.; Zhao, H.; Damon, I.K. GC Content-Based Pan-Pox Universal PCR Assays for Poxvirus Detection. *J Clin Microbiol* **2010**, *48*, 268-276, doi:https://doi.org/10.1128/jcm.01697-09.

49. Okoh, G.s.R.; Lockhart, M.; Grimsey, J.; Whitmore, D.; Ariel, E.; Butler, J.; Horwood, P.F. Development of subfamily-based consensus PCR assays for the detection of human and animal herpesviruses. *Eur J Clin Microbiol Infect Dis* **2023**, *42*, 741-746, doi:https://doi.org/10.1007/s10096-023-04605-w.

50. VanDevanter, D.R.; Warrener, P.; Bennett, L.; Schultz, E.R.; Coulter, S.; Garber, R.L.; Rose, T.M. Detection and analysis of diverse herpesviral species by consensus primer PCR. *J Clin Microbiol* **1996**, *34*, 1666-1671, doi: https://doi.org/10.1128/jcm.34.7.1666-1671.1996.
