# Supplementary material for: Chronic Folliculitis Associated with Ovine gammaherpesvirus 2-Induced Infections in Dairy Cows from Southern Brazil
Source: Animals (Basel). 2025 Oct 1;15(19):2883. doi: 10.3390/ani15192883 (PMC12523531; doi:10.3390/ani15192883)
Supplement: Supplementary file 1 [file animals-15-02883-s001.zip › Supplementary Figure S1.pdf]

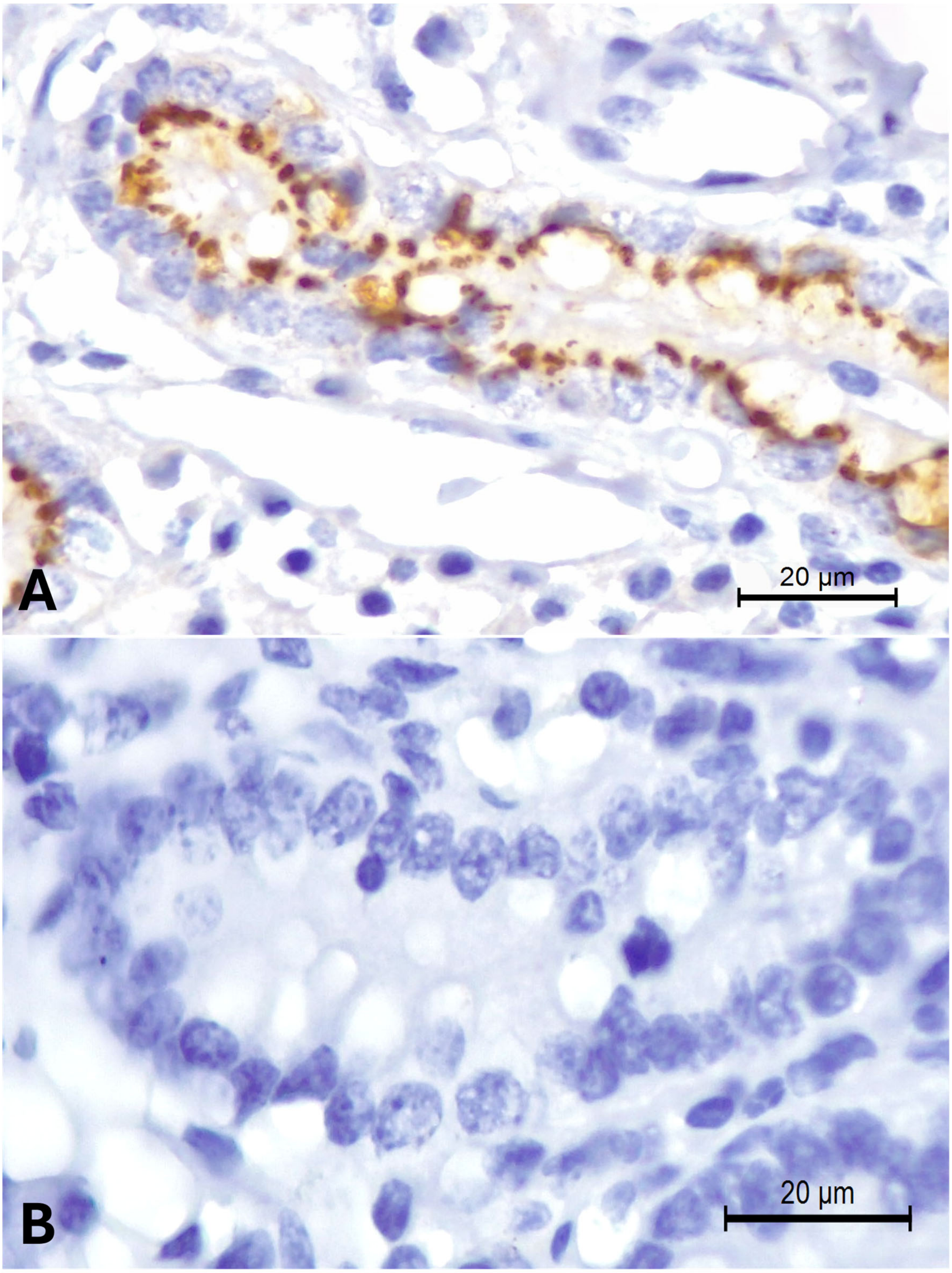

Supplementary Figure S1. Positive (A) and negative (B) controls used in all immunohistochemical assays with the 15A-IHC assay. Immunoperoxidase counterstained with Hematoxylin. Bars, 20 µm.
